# Development of a CAPS Marker and a LAMP Assay for Rapid Detection of *Xylella fastidiosa* Subsp. *multiplex* and Differentiation from *X. fastidiosa* Subsp. *fastidiosa* on Blueberry

**DOI:** 10.3390/ijms23041937

**Published:** 2022-02-09

**Authors:** Sumyya Waliullah, Dario Di Genova, Jonathan E. Oliver, Md Emran Ali

**Affiliations:** 1 Department of Plant Pathology, University of Georgia, Tifton, GA 31793, USA; Sumyya.Waliullah@uga.edu (S.W.); jonathanoliver@uga.edu (J.E.O.); 2 Department of Crop and Soil Sciences, University of Georgia, Tifton, GA 31793, USA; dario.digenova.libero@gmail.com; 3 Department of Agronomy, Food, Natural Resources, Animals and Environment, University of Padova, 35020 Legnaro, Italy

**Keywords:** bacterial leaf scorch (BLS), blueberry, subspecies detection, CAPS marker, LAMP

## Abstract

Bacterial leaf scorch (BLS), caused by *Xylella fastidiosa* (*Xf*), is a prevalent disease of blueberries in the southeastern United States. Initially, this disease was reported to be caused by *X. fastidiosa* subsp. *multiplex* (*Xfm*). However, a recent survey revealed the presence of another subspecies, *X. fastidiosa* subsp. *fastidiosa* (*Xff*), within naturally infected blueberry plantings in Georgia. Since knowledge regarding the origins of isolates causing *Xf* outbreaks can impact management recommendations, a routine method for identifying the pathogen at the subspecies level can be beneficial. Several detection strategies are available to identify *Xf* infection at the subspecies level. However, none of these have been developed for the routine and rapid differentiation of the blueberry-infecting *Xf* subspecies. Here, we developed two separate straightforward and rapid detection techniques, a cleaved amplified polymorphic sequence (CAPS) marker, and a loop-mediated isothermal amplification (LAMP) assay, targeting the RNA polymerase sigma-70 factor (*rpoD*) gene sequence of *Xfm* to discriminate between the two *Xf* subspecies infecting blueberry. With the CAPS marker, specific detection of *Xfm* isolates was possible from pure cultures, inoculated greenhouse-grown plant samples, and field infected blueberry samples by restriction digestion of the *rpoD* gene PCR product (amplified with primers RST31 and RST33) using the *BtsI* enzyme. The LAMP assay allowed for specific real-time amplification of a 204-bp portion of the *Xfm* *rpoD* gene from both pure bacterial cultures and infected plant material using the Genie^®^ III system, a result further affirmed by gel electrophoresis and SYBR™ Green I DNA staining for visual observation. These detection strategies have the potential to greatly aid existing diagnostic methods for determining the distribution and prevalence of these *Xf* subspecies causing bacterial leaf scorch (BLS) in blueberries in the southeastern United States.

## 1. Introduction

*Xylella fastidiosa* (*Xf*) is a Gram-negative, slow growing, and fastidious bacterium in the family *Xanthmonadaceae.* It is a widely distributed plant pathogen which can colonize the xylem of many different plant species, causing a variety of diseases. At least 595 plant species from 275 genera and 85 families have been reported to be infected with *Xf* or *Xylella taiwanensis* [1,2]. However, *Xf* does not appear to cause disease in most of these plant species, and symptoms and diseases caused by *Xf* vary among hosts. The most notorious diseases caused by this pathogen include Pierce’s disease (PD), citrus variegated chlorosis (CVC), phony peach disease (PPD), and several leaf scorch diseases such as almond leaf scorch (ALS), oleander leaf scorch (OLS), coffee leaf scorch (CLS), mulberry leaf scorch (MLS), olive quick decline syndrome (OQDS), and plum leaf scald (PLS) [3,4,5,6]. Another disorder caused by *Xf*, named BLS, was first observed in 2004 affecting southern highbush (SHB) blueberry cultivars in the state of Georgia [7] (U.S.A.) and later in Florida [8]. Disease symptoms of BLS include initial marginal chlorosis of older leaves followed by severe leaf scorch, partial defoliation, severely reduced vegetative growth with reduced numbers of flower buds, dieback, and ultimately death of the plant [7,8,9]. Diseases caused by *Xf* are spread from plant to plant via insect transmission [10], and also by vegetative propagation [11]. Among the insect vectors of *Xf*, glassy-winged sharpshooter (*Homalodisca vitripennis*) is the most common vector found in blueberry plantings in the southeastern United States [12]. 

*Xf* is a genetically diverse species that appears to have originated in the Americas [11], and several distinct subspecies have been described, including *fastidiosa, multiplex, pauca, sandyi, morus,* and *tashke* [13]. This subspecies classification was proposed based on genetic characterization of specific k-mers within the 16S rRNA gene, the 16S-23S intergenic spacer region, and multilocus sequence typing analyses (MLSTs) [10,13,14,15]. Among these subspecies, three (*fastidiosa, multiplex,* and *pauca*) are well-characterized as the causes of damaging diseases on numerous hosts worldwide, while knowledge regarding the other subspecies (*sandyi*, *morus*, and *tashke*) appears to be more limited [13]. Furthermore, it is hard to rule out the existence of additional uncharacterized subspecies, as most of the studies on genetic diversity were carried out on cultivated crops, as well as wild grasses, sedges, and forest trees [1].

Though the actual mechanism of host plant-pathogen specificity is yet to be determined, *Xf* subspecies show host specificity, and it is presumed that genetic variation among subspecies is a determinant of host range. PD of grapevines is caused by *Xf* subsp. *fastidiosa* (*Xff*) while *Xf* subsp. *multiplex* (*Xfm*) causes leaf scorch diseases on multiple plant hosts, including blueberry, almond, oak, and peach in North America [11,16,17]. Additionally, *Xf* subsp. *pauca* (*Xfp*), causes CVC and CLS in Brazil and OQDS in Italy, while *Xf* subsp. *sandyi* (*Xfs*) causes OLS [11,18]. Cross-inoculation of *Xf* isolates from one host to another have often failed to infect the other plant [19]. Failure of *Xfm* isolates from peaches to infect grapevine and *Xff* isolates from grapevine to infect peaches provide some examples [19]. Similarly, *Xfs* isolates from oleander do not infect grapes and *Xff* isolates from grapes do not infect oleander [19]. However, reports suggest that the host range of *Xf* isolates can be extended upon disruption of the cell-to-cell signaling-based gene regulation system [20]. A recent study by Saponari et al. [3] showed that *Xfp* strain De Donno could infect olive, oleander, and myrtle-leaf milkwort in greenhouse conditions. Infection of the same plant host with multiple *Xf* subspecies has also been reported. For example, ALS is reported to be caused by isolates from both *Xfm* and *Xff* subspecies [4,19].

Initially, the *Xf* isolates obtained from BLS diseased blueberry plants were entirely identified as *Xfm* [14,15,21,22]. However, in addition to *Xfm*, recent studies showed that southern highbush blueberry plants can also be infected with *Xff* isolates under greenhouse and field conditions [16,17]. Upon genetic characterization of the *rpoD* gene and utilizing MLST approaches, Di Genova et al. [17] reported that isolates from both subspecies *Xfm* and *Xff* could be found naturally infecting blueberry plantings in southeastern Georgia. Since isolates from these two different subspecies have variable virulence and pathogenicity [16,23], appropriate management recommendations may vary depending on the primary subspecies involved. As such, it is important to understand the distribution and prevalence of these subspecies in blueberry and have precise and prompt methods to differentiate the isolates of *Xfm* and *Xff* affecting this host.

There are several PCR-based detection techniques available to characterize and differentiate between *Xf* subspecies and strains. PCR-RFLP and RAPD are widely used molecular techniques to identify strains and pathotypes of *Xf* [24,25,26]. In previous studies, using a series of specific PCR primers targeting multiple genes and subsequent sequencing of PCR or PCR-RAPD products, several host-specific *Xf* strains including CVC, OLS, MLS, and OQDS strains were detected [23,27,28,29,30]. Nested-PCR (N-PCR), a PCR derivative with increased sensitivity, was applied to identify several strains of *Xf* from American elm, grapevine tissues, citrus plants and sharpshooter leafhoppers [31,32,33]. Exploiting single-nucleotide polymorphisms (SNPs) of 16S rDNA, gyrase B subunit (*gyrB*) and HL hypothetical protein genes, a genetic diversity and phylogenetic analysis based on PCR and subsequent sequencing was carried out on *Xf* from Apulian olive trees revealing the subspecies identity as *pauca* [34]. qRT-PCR (SYBR green or TaqMan) is also a common molecular technique employed to discriminate and identify *Xf* strains causing PD, ALS, OLS, and CVC [35,36,37,38]. A digital droplet PCR assay was utilized to detect *Xf* by adapting a qPCR assay from Harper et al. [39], which targeted the 16S rRNA processing protein gene (*rim*) of the pathogen [40]. In addition, another novel technique, loop-mediated isothermal amplification (LAMP), has also been used to detect *Xf*, as it is a good alternative to PCR that is straightforward and rapid with higher specificity and sensitivity [39,41,42]. However, none of these assays were specifically designed to identify and discriminate between the *Xff* and *Xfm* isolates infecting blueberry.

Therefore, we developed two molecular techniques (i.e., a cleaved amplified polymorphic sequence (CAPS) marker and a LAMP assay targeting the RNA polymerase sigma-70 factor (*rpoD*) gene of *Xfm*) to identify and differentiate between *Xff* and *Xfm* isolates from blueberries. The aim of this study was to develop and validate simple methods to distinguish between *Xff* and *Xfm* isolates efficiently and rapidly. Unlike other methods for subspecies differentiation, the CAPS marker technique does not require the use of multiple PCR primers or subsequent sequencing [43,44], and LAMP is a unique approach widely used for plant pathogen diagnosis for its simplicity, specificity, and sensitivity [42,45,46]. In this study, we were able to identify and differentiate between *Xff* and *Xfm* isolates from blueberry using these two separate assays. These assays have the potential to enhance existing procedures for detecting the subspecies of the pathogen, greatly aiding investigations of *Xf* spread and isolate diversity which will help shape management recommendations for BLS in blueberry production in the southeastern United States.

## 2. Results

### 2.1. Detection and Differentiation of Xfm from Xff Using CAPS Marker

**Figure 1:**
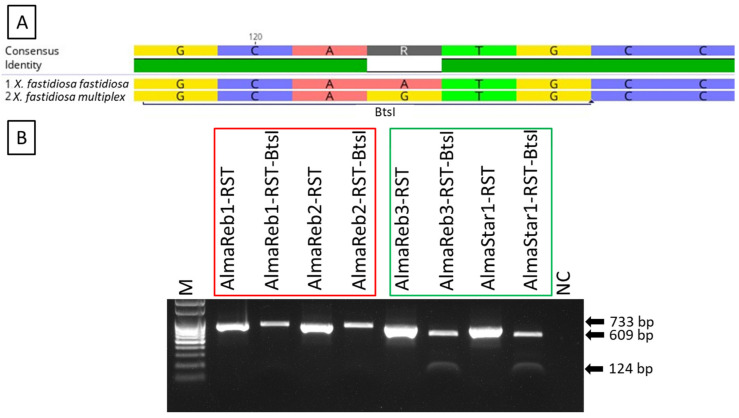
*BtsI* restriction digestion of *X. fastidiosa* RST PCR products from pure bacterial cultures of blueberry isolates from both subspecies. (**A**) Partial sequence showing the *BtsI* restriction site in aligned DNA using consensus sequences from *X. fastidiosa* subsp. *multiplex* and *X. fastidiosa* subsp. *fastidiosa* (for more information see Supplementary Figure S1). (**B**) Agarose gel electrophoresis of *BtsI* restriction digestion fragments of the RST PCR products from DNA extracted from pure cultures of the respective isolates of *X. fastidiosa* subsp. *multiplex* and *X. fastidiosa* subsp. *fastidiosa*. The red box indicates *X. fastidiosa* subsp. *fastidiosa* isolates and the green box indicates *X. fastidiosa* subsp. *multiplex* isolates. “-RST” indicates the undigested product, and “-RST-BtsI” indicates the PCR product after use of the *BtsI* restriction enzyme. M, 100 bp ladder marker; NC, nuclease-free H_2_O used as the negative control. Black arrows indicate the product size in base pairs. Bands of 609-bp and 124-bp can be observed after *BtsI* digestion of the RST PCR products from pure cultures of *X. fastidiosa* subsp. *multiplex* isolates.

**Figure 2:**
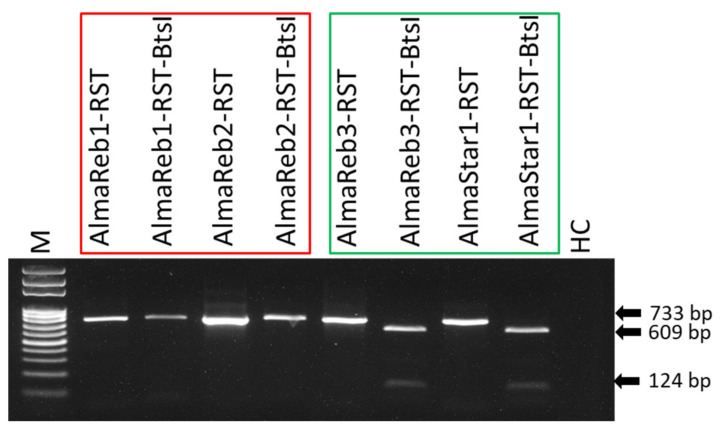
*BtsI* restriction digestion of *X. fastidiosa* subsp. *multiplex* RST PCR products from infected greenhouse-grown blueberry plant samples. Agarose gel electrophoresis of *BtsI* restriction digestion fragments of the RST PCR products from DNA extracted from greenhouse-grown plants infected with the respective isolates of *X. fastidiosa* subsp. *multiplex* and *X. fastidiosa* subsp. *fastidiosa*. The red box indicates plant samples infected with *X. fastidiosa* subsp. *fastidiosa* isolates and the green box indicates plants samples infected with *X. fastidiosa* subsp. *multiplex* isolates. “-RST” indicates the undigested product, and “-RST-BtsI” indicates the PCR product after use of the *BtsI* restriction enzyme. M, 100 bp ladder marker; HC, healthy SHB plant sample as healthy control. Black arrows indicate the product size in base pairs. Bands of 609-bp and 124-bp can be observed only after *BtsI* digestion of the RST PCR products from plants infected with *X. fastidiosa* subsp. *multiplex* isolates.

Use of the *BtsI* restriction enzyme and a unique restriction site in *Xfm* (Figure 1A, Supplementary Figure S1) was evaluated for distinguishing between *Xfm* and *Xff* isolates from blueberry. The enzyme was used to cleave RST PCR products (abbreviated name of PCR product amplified using primer set RST31/RST33, Supplementary Table S1) from pure bacterial cultures and greenhouse-grown blueberry plants inoculated with *Xfm* (AlmaReb3 and AlmaStar1) or *Xff* (AlmaReb1 and AlmaReb2) isolates. Regardless of the origin of the PCR products, restriction digested PCR fragments were only detected from *Xfm* samples, while polymorphisms were not detected with *BtsI* digests from *Xff* samples (Figure 1 and Figure 2). Presence of the cleaved 609-bp and 124-bp bands from the 733-bp RST PCR products indicated the presence of *Xfm* isolates in both experiments (Figure 1 and Figure 2).

### 2.2. Detection and Differentiation of Infected Field Samples Using CAPS Marker

**Figure 3:**
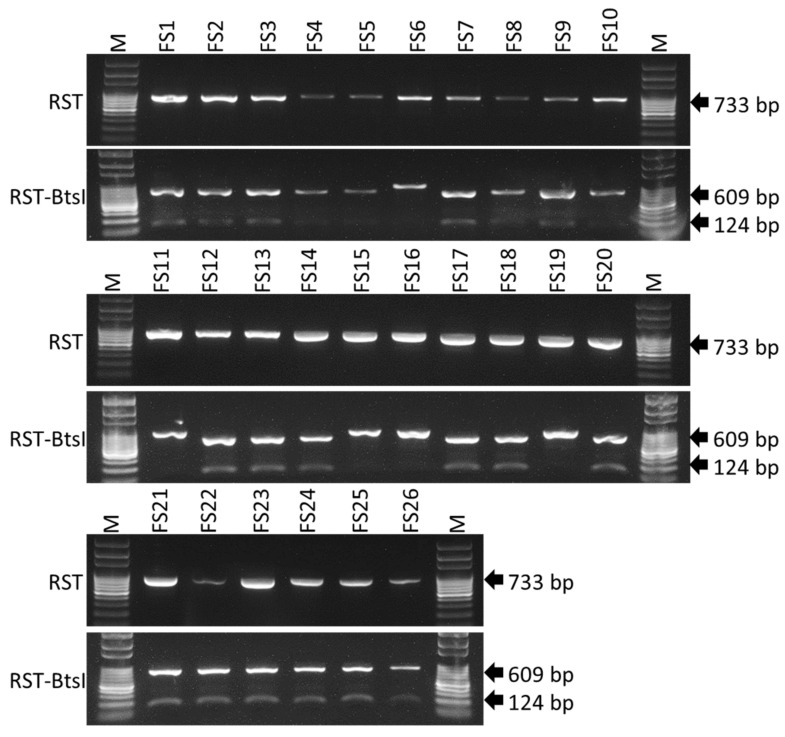
*BtsI* restriction digestion of *X. fastidiosa* subsp. *multiplex* RST PCR products from infected field plant samples. FS1 to FS26: undigested (upper lane on gel photographs) and *BtsI*-digested (lower lane on gel photographs) RST PCR products of 26 collected field samples positive for *X. fastidiosa*. M = 100 bp ladder marker. Black arrows indicate the product size in base pairs.

A total of 26 *Xf* positive SHB field samples (Table 1) were screened by CAPS marker for subspecies detection and differentiation. Upon digestion of RST PCR products with *BtsI,* 21 cleaved PCR products were observed following gel electrophoresis (Figure 3). The separated 609-bp and 124-bp bands indicate infection with *Xfm*, whereas the other 5 undigested PCR products indicate infection with *Xff*. The subspecies identity of the infected field samples was further affirmed by direct sequencing of the PCR product (Table 1).

### 2.3. LAMP Condition Optimization for Xfm Detection

**Figure 4:**
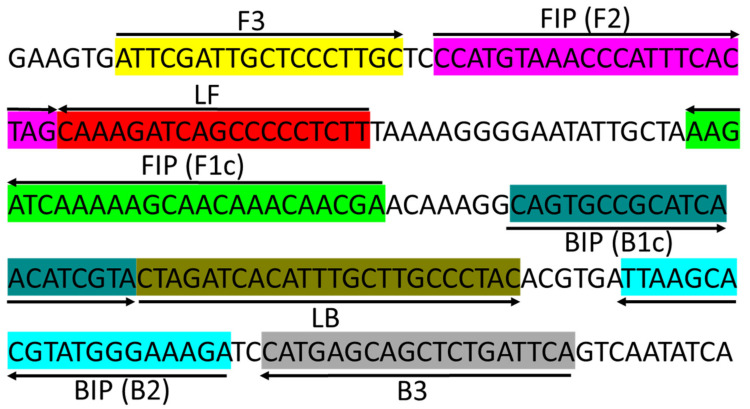
Location and partial sequence of loop-mediated isothermal amplification (LAMP) primer sets (F3, B3, FIP [F1c-F2], and BIP [B1c-B2]) targeting *X. fastidiosa* subsp. *multiplex* RNA polymerase sigma-70 factor *rpoD* locus. FIP is a hybrid primer consisting of the F1c sequence and the F2 sequence, BIP is a hybrid primer consisting of the B1c sequence and the B2 sequence. Arrows indicate the extension direction.

**Figure 5:**
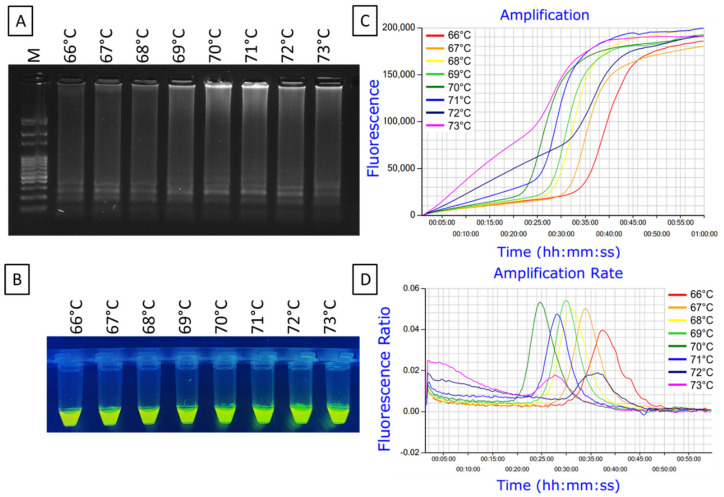
A gradient LAMP for the optimization of reaction temperature for *X. fastidiosa* subsp. *multiplex* LAMP. Gradient LAMP was set from 66 to 73 °C. Results were analyzed by: (**A**) agarose gel electrophoresis, (**B**) SYBR™ green 1 DNA gel staining agent for visual amplification and (**C**) real-time amplification by Genie^®^ III. (**D**) Amplification rate shows the peak value (min:sec) of the amplified curves. Lane M = 100 bp DNA ladder. The fluorescence green-colored products from the positive LAMP reactions could be visualized after adding SYBR™ green 1 DNA gel staining agent with the LAMP amplified end product under UV light.

The optimal reaction temperature for the *Xfm* LAMP assay was determined using 0.1 ng/µL DNA extracted from a pure culture of *Xfm* isolate AlmaReb3. The newly synthesized LAMP primer sets including F3, B3, FIP [F1c-F2], BIP [B1c-B2], LF and LB designed by Primer Explorer version 5 (Figure 4, Supplementary Table S1) using the RNA polymerase sigma-70 factor *rpoD* locus from *Xfm* sequence (Figure 1, Supplementary Table S1) were used to optimize the temperature conditions. Based on the previous studies and this study, the optimum 10× formulation of the LAMP primer mix was determined to be: 0.2 μM each of primers F3 and B3, 0.8 μM each of primers LF and LB, and 1.6 μM each of primers FIP and BIP [45,46]. To rule out the optimum reaction temperature, a gradient LAMP was performed from 66 to 73 °C using the 10× formulation of the six LAMP primers with 0.1 ng/µL of diluted *Xfm* pure culture DNA. A reaction conducted at 70 °C had the optimal outcome with the shortest peak time (Ti_amp_) of 24 min and 30 sec and a melting curve with a specific peak at 83.6 °C (Figure 5, Supplementary Table S2). In our previous studies, we observed that a 60 min reaction time is adequate for LAMP amplification [45,46]. Therefore, 60 min was determined to be the optimal reaction time to complete amplification of *Xfm*. Thus, the optimal reaction conditions for the LAMP were determined to be 70 °C for 60 min.

### 2.4. Amplification of Xfm Using LAMP Assay

**Figure 6:**
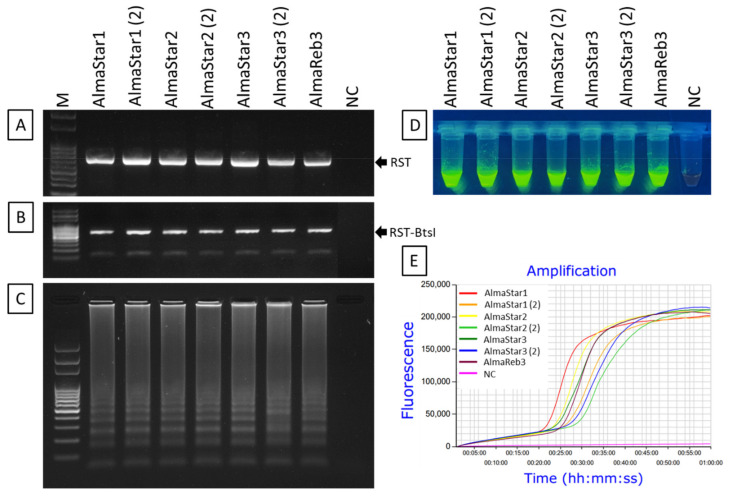
LAMP detection of *X. fastidiosa* subsp. *multiplex* from DNA derived from pure bacterial cultures. (**A**,**B**). Amplified RST PCR products were digested with *BtsI* restriction enzyme for subspecies confirmation. Successful LAMP amplification of those isolates was visualized using: (**C**) agarose gel electrophoresis, (**D**) colorimetric visual inspection by adding SYBR™ green 1 DNA gel staining agent, and (**E**) real-time amplification using Genie^®^ III. All isolates depicted belong to *X. fastidiosa* subsp. *multiplex*. The (2) following the isolate name indicates that DNA from a second subculture of the same isolate was tested. M, 100 bp DNA ladder; and NC, nuclease-free H_2_O used as the negative control. The fluorescent green-colored products for each isolate, excluding the negative control, could be visualized after adding SYBR™ green 1 DNA gel staining agent under UV light.

To examine the applicability of the LAMP assay for the detection of *Xfm*, the method was evaluated using 0.1 ng/µL of DNA extracted from pure bacterial cultures of *Xfm* isolates AlmaReb3, AlmaStar1, AlmaStar2, and AlmaStar3. DNA extracted from a second subculture of AlmaStar1, AlmaStar2, and AlmaStar3 was also utilized. Subspecies identities for each pure culture isolate were confirmed by CAPS marker (Figure 6A,B). LAMP primer sets specifically designed targeting the variations among *Xfm* and *Xff rpoD* sequences (Supplementary Figure S3) could detect *Xfm* isolates (Figure 6). All seven cultures from *Xfm* isolates demonstrated positive amplification of LAMP with gel electrophoresis (Figure 6C), SYBR™ Green I nucleic acid gel stain (Figure 6D), and Genie^®^ III (OptiGene, Horsham, WS, UK) real-time amplification system (Figure 6E). No amplification was observed in the negative control (Figure 6).

### 2.5. Sensitivity of LAMP Detection of Xfm

**Figure 7:**
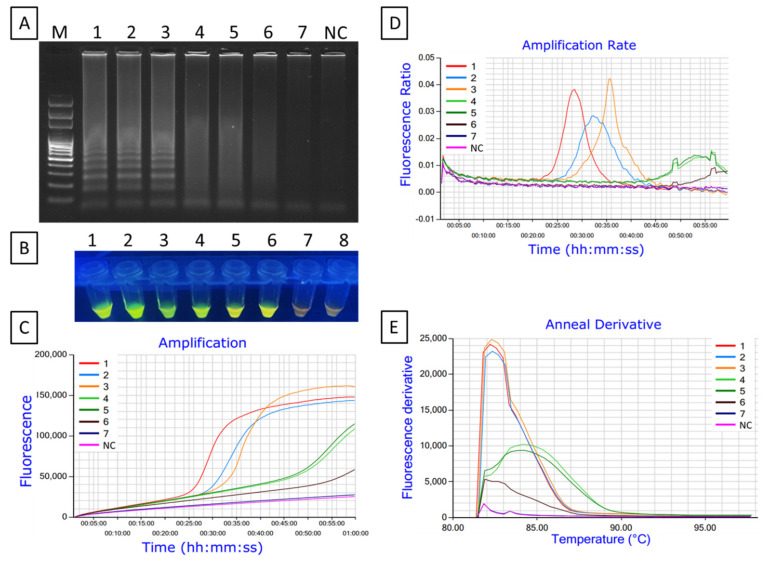
Sensitivity of the LAMP assay for the detection of *X. fastidiosa* subsp. *multiplex* using DNA from *Xfm* isolate AlmaReb3. Amplified LAMP products were analyzed using: (**A**) agarose gel electrophoresis, (**B**) visual inspection with SYBR™ green 1 DNA gel staining agent, and (**C**) real-time amplification using Genie^®^ III. (**D**, **E**) amplification rate and anneal derivative curves obtained from Genie^®^ III. Here, 1 to 7: 100 pg/µL to 0.0001 pg/µL of *Xfm* DNA. M, 100 bp DNA ladder; NC, nuclease-free H_2_O used as a negative control. The fluorescent green-colored products could be visualized after adding SYBR™ green 1 DNA gel staining agent to the end product under UV light while negative reactions remained orange in color.

Sensitivity of the LAMP primers was assessed using a series of 10-fold dilutions of total DNA (10 to 10^−7^ ng/μL or 10 ng/μL to 0.1 fg/μL) extracted from *Xfm* isolate AlmaReb3. The amplification result from Genie^®^ III demonstrated that LAMP amplified products were readily detectable until 1 pg/µL with a true amplification curve, higher amplification rate, and anneal derivative (Figure 7C–E). Genie^®^ III amplified LAMP product was further confirmed by gel electrophoresis and SYBR™ Green I DNA gel staining agent (Invitrogen, Waltham, MA, USA) for visual observation. Ladder-like amplified LAMP product was only visible up to 1 pg/µL of DNA in the gel electrophoresis photograph, whereas lower DNA concentrations showed a smear or no amplified product (Figure 7A). A similar data outcome was observed with adding SYBR™ green 1 DNA gel staining agent (Invitrogen) under UV light, where bright green fluorescent colored products were visible with higher DNA concentrations and weak or negative reactions turned gradually to orange in color (Figure 7B). Therefore, the sensitivity of the LAMP assay was determined to be 1 pg/µL of DNA (Figure 7, Supplementary Table S3).

### 2.6. Specificity of LAMP Detection of Xfm

**Figure 8:**
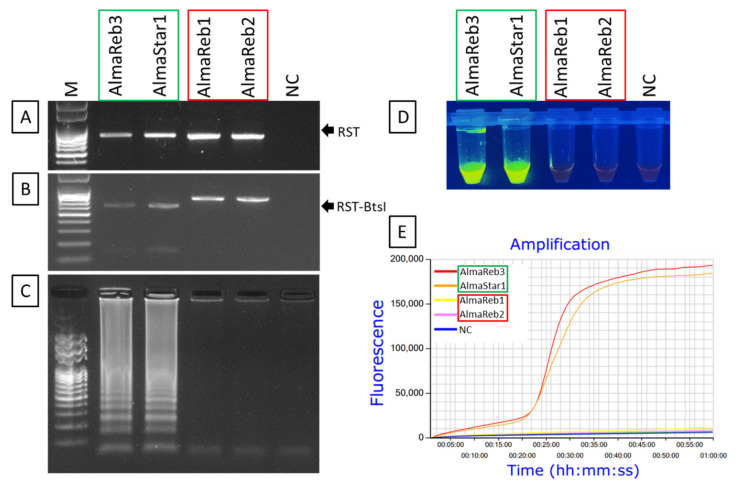
Specificity of the LAMP assay for the detection of *X. fastidiosa* subsp. *multiplex* using DNA from pure cultures of blueberry *X. fastidiosa* isolates from both subspecies. The red box indicates *X. fastidiosa* subsp. *fastidiosa* isolates and the green box indicates *X. fastidiosa* subsp. *multiplex* isolates. (**A**,**B**) Amplified RST PCR products were digested with *BtsI* restriction enzyme for subspecies confirmation. Amplified LAMP products were analyzed using: (**C**) agarose gel electrophoresis, (**D**) visual inspection with SYBR™ green 1 DNA gel staining agent, and (**E**) real-time amplification using Genie^®^ III. M, 100 bp DNA ladder; NC, nuclease-free H_2_O as a negative control. The fluorescent green-colored products could be visualized after adding SYBR™ green 1 DNA gel staining agent to the end product under UV light while negative reactions remained orange in color.

**Figure 9:**
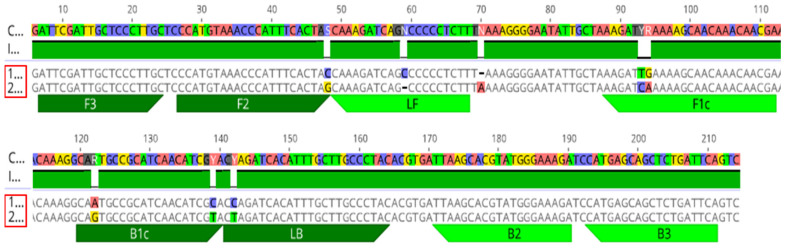
An alignment showing SNPs, insertion and deletion mutations (colored letters) for *X. fastidiosa* subsp. *multiplex* LAMP primer binding sites versus *X. fastidiosa* subsp. *fastidiosa* in *rpoD* consensus sequences. The red box is showing the numbers, where 1: *X. fastidiosa* subsp. *fastidiosa*, 2: *X. fastidiosa* subsp. *multiplex*. Here: C, consensus; I, identity. For more information, see Supplementary Figure S3.

Specificity of the LAMP primers was assessed using 0.1 ng of DNA from *Xfm* blueberry isolates AlmaReb3 and AlmaStar1 and *Xff* blueberry isolates AlmaReb1 and AlmaReb2. For further confirmation, RST PCR products (Figure 8A) were digested with the *BtsI* restriction enzyme (Figure 8B). An additional specificity test was carried out with 7 isolates (3 *Xff* and 4 *Xfm*) to further affirm LAMP primers amplification specificity (Supplementary Figure S2). After LAMP amplification, only *Xfm* isolates showed positive reaction by LAMP, while no amplification by the LAMP assay was observed from *Xff* isolates or the negative control (Figure 8C–E). Results were confirmed using three different detection strategies including agarose gel image analysis (Figure 8C), SYBR™ green-based UV fluorescence (Figure 8D) and Genie^®^ III amplification curve analysis (Figure 8E). All three detection strategies showed a positive reaction only for DNA from *Xfm* isolates, but not from *Xff* isolate DNA or the negative control (Figure 8). The results indicated that the LAMP assay could distinguish *Xfm* isolates from blueberry from *Xff* isolates from blueberry. An alignment of the LAMP primer binding sites in *Xfm* versus *Xff* illustrates how the LAMP assay could specifically distinguish between these two subspecies (Figure 9, Supplementary Figure S3). The nucleotide differences between *Xfm* and *Xff* within the targeted *rpoD* region illustrate how specific detection of *Xfm* occurs (Figure 9, Supplementary Figure S3).

### 2.7. Detection of Xfm from Greenhouse-Grown and Infected Field Samples Using Probe-Based LAMP

**Figure 10:**
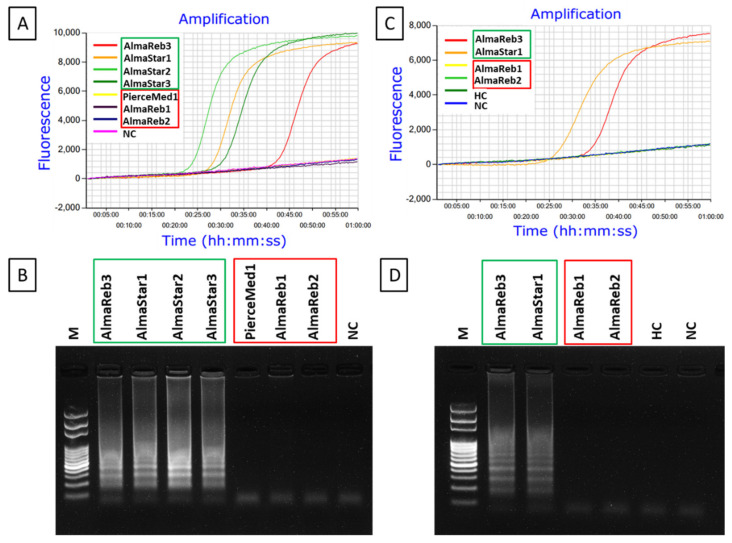
Detection of *X. fastidiosa* subsp. *multiplex* from known bacterial cultures (**A**,**B**) and infected greenhouse-grown samples (**C**,**D**) by probe-based LAMP. Successful LAMP amplification of *Xfm* isolates or infected plant tissues were visualized using real-time amplification with Genie^®^ III (**A**,**C**) and with agarose gel image (**B**,**D**). The red box indicates *X. fastidiosa* subsp. *fastidiosa* (*Xff*) isolates or infected greenhouse-grown samples and the green box indicates *X. fastidiosa* subsp. *multiplex* (*Xfm*) isolates or infected greenhouse-grown samples. Here: M, 100 bp DNA ladder; NC, nuclease free H_2_O used as the negative control.

**Figure 11:**
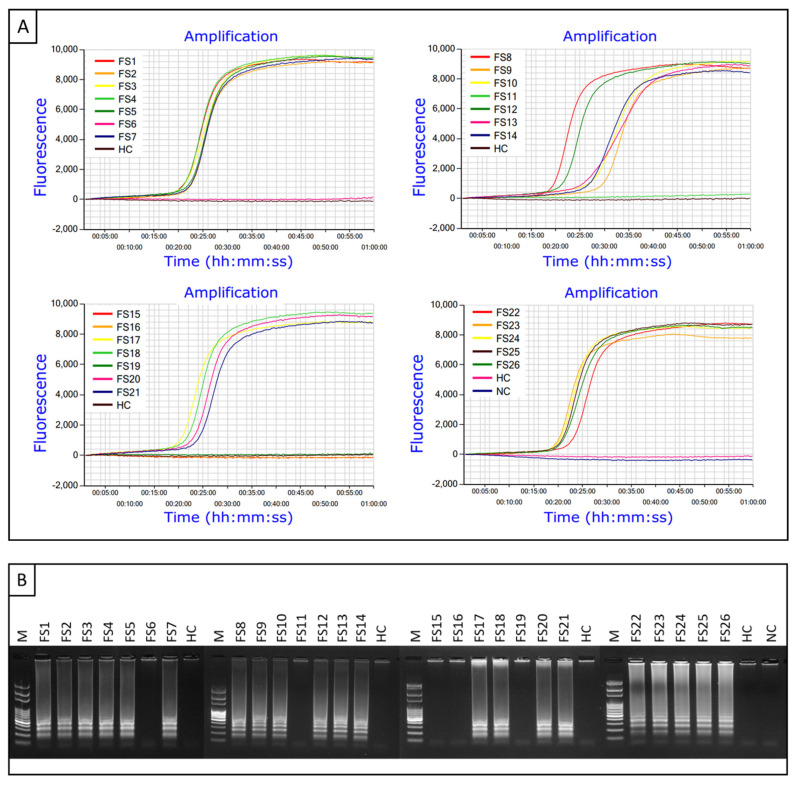
Detection of *X. fastidiosa* subsp. *multiplex* from infected field plant samples using probe-based LAMP. Successful LAMP amplification of *Xfm*-infected plant tissues was visualized using real-time amplification with Genie^®^ III (**A**) and with agarose gel imaging (**B**). Here, FS1 to FS26: 26 collected field infected samples positive for *X. fastidiosa*. M, 100 bp ladder marker; HC, healthy blueberry leaf tissues as healthy control; NC, nuclease free H_2_O used as the negative control.

The specificity of the LAMP primers was also tested with the same greenhouse and infected field leaf samples that were assessed here with the CAPS marker (Figure 2 and Figure 3). However, preliminary results using the LAMP assay on infected blueberry leaf samples were not consistent. To enable LAMP amplification of infected host samples, a Florescence resonance energy transfer (FRET)-based assimilating probe [47] was developed that enabled the detection of *Xfm* directly from infected blueberry samples using LAMP (Supplementary Figure S5, Supplementary Table S1, Figure 10 and Figure 11). The reaction conditions for the probe-based LAMP were optimized for reaction temperature and probe/primer mixture volume using 0.1 ng/µL DNA extracted from *Xfm* isolate AlmaReb3. The optimized temperature for the probe-based LAMP was determined with a gradient LAMP, where the fastest LAMP amplification was obtained at 70 °C (Supplementary Figure S6A,B; Supplementary Table S4). The primer/probe mixture volume of 2.5 µL with a concentration of 0.2 μM of F3 and B3, 1.6 μM of FIP and BIP, 0.1 μM of the quench (Q) strand and 0.2 μM of the FAM-tagged LB assimilating probe with 0.8 μM of LF primer was considered as an optimized reaction condition, where no false amplification with negative control occurred and efficient amplification with the positive control manifested by the observation of a true sigmoid amplification curve (Supplementary Figure S6C,D). The sensitivity test with 10-fold serially diluted DNA (10 to 10^−7^ ng/μL or 10 ng/μL to 0.1 fg/μL) from AlmaReb3 revealed that the detection limit of the probe-based LAMP was at 0.1 pg/µL (Supplementary Figure S7, Supplementary Table S5). However, a true sigmoid amplification curve was only observed down to 10 pg/µL with a brighter ladder-like gel electrophoresis band (Supplementary Figure S7). The optimized FAM-tagged probe/primer set was then primarily assessed with culture grown *Xfm* isolates as well as with greenhouse-grown infected plant samples. The initial experiments demonstrated that the probe-based LAMP assay was successfully able to selectively amplify *Xfm* from laboratory-grown isolates and infected plant samples without any cross-reactions (Figure 10). The 26 *Xf* positive SHB field samples (Table 1) that were screened by CAPS marker for subspecies detection and differentiation were also screened with the modified probe-based LAMP assay. Among those infected field samples, 21 LAMP amplified samples were *Xfm* infected SHB leaf samples, which matched perfectly with the results from CAPS marker and direct sequencing of the RST PCR product (Figure 11, Table 1).

## 3. Discussion

Bacterial leaf scorch (BLS) caused by *Xf* is a major concern for blueberry production in the southeastern United States. As *Xf* is a genetically diverse species with distinct subspecies that vary in host range and pathogenicity, management of this pathogen is often challenging. So far, two subspecies of *Xf* (i.e., *Xfm* and *Xff*) are reported to infect blueberry and cause BLS in blueberry plantings in southeastern Georgia [16,17]. In order to characterize the prevalence of these subspecies in blueberry and understand the origins of the isolates causing BLS in blueberry plantings, it is essential to precisely identify and differentiate between infections with these respective subspecies in Georgia blueberry production areas.

Development of a single test that can be used in all conditions to identify these subspecies has not previously been reported. Combinations of multiple approaches for subspecies detection are described in several studies and vary in sensitivity, specificity and ease of use. A multiprimer PCR assay with three different targets was developed by Hernandez-Martinez et al. [48] to differentiate strains of *X. fastidiosa* infecting grape, almonds and oleander. Strains from three different subspecies (i.e., *Xfm*, *Xff*, and *Xfs*) were detected using this strategy. In another study carried out by Melanson et al. [49] on pecan, a combination of a multiprimer PCR assay with two other PCR-based techniques (i.e., enterobacterial repetitive intergenic consensus (ERIC)-PCR and repetitive extragenic palindromic (REP)-PCR was used to classify *Xf* strains. In addition, with those PCR assays, amplification and sequence analysis of the 16S-23S ITS and *pglA* gene was performed to confirm that *Xf* strains infecting pecan belong to subsp. *multiplex* [49]. Besides PCR based assays, a novel tetraplex qPCR assay was developed recently to identify *Xf* subspecies from a wide variety of hosts [50]. However, these techniques were not developed to differentiate between the *Xff* and *Xfm* strains present in blueberry. Recently, Faino et al. [51] leveraged Oxford Nanopore Technologies (ONT) MinION platform to detect and identify *Xf* at the subspecies and Sequence Type (ST) level. However, regardless of its advantages for detecting pathogen identity at subspecies level, ONTs MinION device has several drawbacks including having a high error rate, requiring frequent replacement of the flow cell, and requiring expertise to handle the data output (since contaminating sequence data could be misleading) [52].

Therefore, we developed two different methods that could be used to precisely identify the two subspecies of *Xf* (i.e., *Xfm* and *Xff*) causing BLS in blueberry from pure cultures or from infected blueberry plant material. A CAPS marker, which does not need any complex equipment or sequence analysis, and a LAMP assay, which is a rapid, sensitive, simple, and portable diagnostic method, were developed in this study. Using a single cut with the *BtsI* restriction enzyme (Figure 1A, Supplementary Figure S1), the CAPS marker could differentiate between the subspecies of pure culture of *Xf* blueberry isolates as well as greenhouse or field-grown plant samples infected with *Xfm* or *Xff* isolates (Figure 1, Figure 2 and Figure 3). The 733-bp PCR product of the *rpoD* gene was cleaved by the *BtsI* enzyme at nt 124 of *Xfm* isolates and *Xfm*-infected samples, whereas PCR products from *Xff* isolates and *Xff*-infected plant samples remained undigested (Figure 1, Figure 2 and Figure 3). In one of our previous studies, we determined the detection limit for the RST PCR primers to be between 2.5 pg and 1.25 pg of *Xf* genomic DNA per reaction [42] and, in the current study, we also observed that the CAPS marker could detect a distinctive PCR product at 2.5 pg of *Xf* genomic DNA per reaction.

In addition to the CAPS marker, a LAMP assay was also developed for subspecies identification. Previously, a novel LAMP assay was developed targeting the 16S rRNA processing protein gene *rim* by Harper et al. [39] to detect *Xf.* That study used 20 isolates of *Xf* from four subspecies of the pathogen [39]. Nonetheless, the aim of that study was to detect *Xf* isolates regardless of subspecies, not to differentiate them based upon subspecies. Here, we designed the LAMP targeting SNPs, insertion and deletion mutations present in *Xfm* versus *Xff* isolates from blueberry (Figure 8, Supplementary Figure S3) to distinguish between blueberry isolates of the two subspecies. The LAMP assay was developed targeting the variations of *Xfm* isolates in *rpoD* sequences compared to *Xff*, since preliminary data revealed *Xfm* isolates as more prevalent in Georgia (Table 1, Figure 11) [17]. Optimization of the reaction temperature using DNA extracted from *Xfm* isolates as templates revealed 70 °C as the optimum annealing temperature for the LAMP assay (Figure 5). The detection limit of the LAMP assay was determined to be 1 pg/µL of *Xfm* DNA (Figure 9, Supplementary Table S3). Successful amplification of *Xfm* isolates was observed (Figure 6) without cross-reaction with *Xff* isolates (Figure 7, Supplementary Figure S2), illustrating the high specificity of the LAMP assay described in our study. Moreover, the amplified products could be visualized using three different detection strategies: (1) agarose gel electrophoresis, (2) adding SYBR^®^ Green I nucleic acid gel staining agent (Invitrogen) to the LAMP amplified end product, and (3) real-time amplification by Genie^®^ III (Figure 6 and Figure 7), which made this assay a good substitute for other complicated and highly instrument-dependent molecular assays. The LAMP assay was also assessed with infected blueberry plant samples. However, as we initially observed inconsistencies among data outcome from the LAMP with plant samples, the LAMP primer sets were modified according to Kubota et al. [47]. Specifically, for use with the other five regular LAMP primers, we modified the backward loop primer (LB) by tagging it with a 6-carboxyl-flurescein (FAM) to act as an assimilating probe with a synthetic quench (Q) strand (Supplementary Figure S5, Supplementary Table S1). The optimum annealing temperature of this modified probe-based LAMP was the same at 70 °C as the regular LAMP (Supplementary Figure S6, Figure 6). However, it should be noted that a decreased sensitivity was observed for the modified LAMP probe/primer sets versus the regular LAMP reaction, where a true amplification curve was observed at 10 pg/µL rather than at 1 pg/µL (Supplementary Figure S7, Figure 7). Nonetheless, disregarding the lower sensitivity, successful detection of the *Xfm* infection from infected SHB blueberry plant samples without cross-reactions proves the field applicability of the modified probe-based LAMP assay (Figure 10 and Figure 11). Thus, both of the LAMP assays can be used based on the aim of differentiating subspecies identity: the regular LAMP assay as a low-cost option to detect *Xfm* from culture grown isolates, and the modified probe-based LAMP assay to detect *Xfm* infection regardless of the origin of the samples (i.e., from both pure culture-grown isolates and infected blueberry plant materials). Future studies could focus on the development of a multiplex LAMP assay or CAPS markers targeting several subspecies of *Xf* in a single reaction to investigate any potential new subspecies that might infect blueberries.

Taken together, these results suggest that the CAPS marker and LAMP assays described herein could discriminate between *Xfm* and *Xff* by restriction digestion or by specific amplification of only *Xfm* isolates, preventing the need for further sequencing. Both of these assays could be used independently or in combination, applicable in various conditions, for the subspecies identification and differentiation of *Xf* isolates infecting blueberries. Accordingly, these assays represent a valuable diagnostic tool for disease surveillance to help characterize the distribution and diversity of *Xf* on a broad scale with higher throughput in blueberry production fields in the southeastern United States.

## 4. Materials and Methods

### 4.1. Bacterial Isolates and Growth Conditions

Seven distinct *Xf* isolates from two different subspecies of *Xylella fastidiosa* (i.e., *X. fastidiosa* subsp. *fastidiosa* (*Xff*) and *X. fastidiosa* subsp. *multiplex* (*Xfm*)) were originally cultured from symptomatic, naturally infected SHB blueberry plantings in Pierce and Bacon County, Georgia [17] and suspended in glycerol for long-term storage (>6 months) at −80 °C. The name, origin, and subspecies identity of each isolate utilized in this study are provided in Table 2. The collected isolates were recovered from long-term storage by plating bacterial suspensions on PW (periwinkle wilt) [53] medium to extract DNA for molecular analysis and to mechanically inoculate greenhouse-grown blueberry plants for further analysis [53,54,55]. Two to three subcultures were used from each isolate to confirm the feasibility of the detection methods developed in this study.

### 4.2. Plant Materials and Tissue Preparation

To test the accuracy of the molecular assays developed in this study on known infected plants, blueberry plant samples inoculated with either *Xff* or *Xfm* isolates were collected from the greenhouse for bacterial subspecies detection and differentiation. The SHB cultivar ‘Rebel’ plants were grown in greenhouse conditions and inoculated with known isolates of *Xf* following the methods as reported by Oliver et al. [17]. Internodes (5–6″ long) from SHB plants with 10 to 12 mature leaves were sampled for molecular analysis. For DNA extraction, leaf samples were surface sterilized with 5% NaOCl solution. Uninoculated and inoculated blueberry plants were used as sources of negative and positive plant tissue controls.

### 4.3. DNA Isolation

The CTAB DNA extraction solution (G-Bioscience, St. Louis, MO, USA) was used for DNA preparations from bacterial cells and infected plant samples from the greenhouse and field with a slight modification to the recommended CTAB protocol by G-Bioscience [45]. Briefly, for DNA extraction from bacteria, inoculum was taken from PW [53] agar medium grown for 7 to 10 days at 28 °C using sterile, disposable inoculating loops. For plant samples, infected leaves were flash-frozen in liquid nitrogen and pulverized using a mortar and pestle. About 100 mg of sample was used for DNA extraction. The collected bacterial cells and 100 mg leaf samples were then re-suspended in 500 μL of CTAB extraction solution containing 1% PVP. Proteins and other cellular components were removed by using CTAB and by chloroform and isopropanol extractions according to the protocol described in Waliullah et al. [45]. The DNA pellet was washed with 500 μL cold 70% ethanol in a final step during the extraction procedure, and nucleic acids were recovered (12,000× *g*, 5 min), resuspended in TE buffer (10 mM Tris–HCl, 0.5 mM EDTA, pH 8), and either used immediately or stored at −20 °C for subsequent use. Total DNA yield and purity were estimated by measuring OD 260 nm and OD 260 nm/280 nm with a NanoDrop spectrophotometer (NANODROP LITE, Thermo Scientific, Waltham, MA, USA).

### 4.4. PCR Amplification of the Xf-Specific rpoD Gene

PCR primer set RST 31/33 targeting RNA polymerase sigma-70 factor (*rpoD*), which has been widely accepted for the detection of the pathogen [55], was used to detect *Xf* from bacterial culture and blueberry leaf samples. PCR reactions were performed on a thermocycler (Bio-Rad-96 well T100™, Bio-Rad, Hercules, CA, USA) using EconoTaq PLUS GREEN 2× Master Mix (Lucigen, Madison, WI, USA) according to the manufacturer’s instructions. For PCR reactions with DNA extracted from pure bacterial cultures, 1 ng of template DNA was used. For the analysis of the experimental samples from the greenhouse and field, 20 ng of DNA was used in each reaction mixture. Each reaction contained the specified amount of template DNA with 0.3 μM of each forward and reverse primer (Supplementary Table S1), 10 μL of 2× Econotaq master mix (Lucigen), and deionized PCR grade water added to reach a final volume of 20 μL. PCR conditions for RST 31/33 were as follows: an initial denaturation step at 95 °C for 2 min followed by 40 cycles of 30 s at 95 °C, 30 s at 58 °C, 45 s at 72 °C and final extension at 72 °C for 5 min. PCR products were checked on a 1.0% Tris-borate-EDTA (TBE) agarose gel. Samples were considered PCR positive when the DNA band of the expected size (733-bp) was clearly visualized after electrophoresis. Amplified products were purified with Quantum Prep^®^ PCR Kleen Spin Columns (Bio-Rad) with a slight modification to the manufacturer’s protocol.

### 4.5. CAPS Marker Development and Analysis of the PCR Product

To identify unique restriction sites between subspecies *Xfm* and *Xff*, all publicly available RNA polymerase sigma-70 factor *rpoD* gene sequences of *Xff* and *Xfm* isolates from blueberry (utilized in this study and from GenBank) were imported into Geneious v10.1.2 (Biomatters Ltd., Auckland, New Zealand) software (Figure 1A, Supplementary Figure S1, Table 2). For sequence alignment, the Align/Assemble > Pairwise/Multiple Align function using “Geneious Alignment” option with default settings were employed. The unique restriction site was identified using “Find Restriction Sites” option with default settings. Commonly used enzymes were searched to identify an appropriate candidate enzyme. The *BtsI* enzyme was selected to differentiate in between the two subspecies, as it has a unique restriction site present in *Xfm* (Figure 1A, Supplementary Figure S1) that is absent in *Xff* isolates from blueberry. To proceed with CAPS marker, 10 µL of RST PCR product purified with Quantum Prep^®^ PCR Kleen Spin Column (Bio-Rad) was digested with 0.5 µL of *BtsI-v2* restriction enzyme (NEB, Beverly, MA, USA) with 2.5 µL of 10× NE Buffer and nuclease-free H_2_O, totaling 25 µL per reaction. The reaction mixture was incubated according to the manufacturer’s protocol. The CAPS reaction products were separated by electrophoresis in 1.0% TBE agarose gels following staining with GelGreen^®^ Nucleic Acid Gel Stain (Biotium, Fremont, CA, USA) and visualized on a UV transilluminator UVP UVsolo touch (Analytik Jena, Upland, CA, USA).

### 4.6. CAPS Analysis of PCR Product from Pure Bacterial Cultures and Infected Plant Samples

RST PCR reactions were performed with the DNA from pure bacterial cultures (Section 4.1) and greenhouse-grown blueberry plants which had previously been inoculated with *Xfm* or *Xff* isolates (Section 4.2). PCR products were digested with the *BtsI-v2* restriction enzyme (NEB) following the methods described above (Section 4.5). Agarose gel electrophoresis was carried out with 5 µL of the undigested and digested CAPS products concurrently.

### 4.7. LAMP Primer Design

The published RNA polymerase sigma-70 factor *rpoD* gene sequences for *Xff* and *Xfm* isolates from blueberry (Table 2) were aligned and searched for SNPs and in/del mutations (Section 4.5, Figure 8, Supplementary Figure S3, Table 2). Two in/del mutations and several other SNPs were found within the region of interest. Therefore, the region with the most variations in *Xfm* (relative to *Xff*) covering 204-bp of the *rpoD* gene was targeted for LAMP primer design using Primer Explorer version 5 (http://primerexplorer.jp/lampv5e/, accessed on 6 February 2022). The primers included two outer primers (F3 and B3), two inner primers (FIP and BIP), and two loop primers (LF, LB). For the FRET-based LAMP assay, besides the other 5 regular LAMP primers described above, the backward loop primer (LB) sequence was modified to generate the assimilating probe with a 6-carboxyl-flurescein (FAM) florescent tag and an associated quencher strand that is displaced by a BHQ -tagged quench (Q) strand during new strand synthesis (Supplementary Figure S5, Supplementary Table S1). Primers were synthesized by Sigma-Aldrich (St. Louis, MO, USA), dissolved in nuclease-free H_2_O (Sigma-Aldrich) to produce 100 µM solutions, and stored at −20°C.

### 4.8. Optimization of LAMP Conditions

To optimize the reaction conditions of LAMP, total DNA extracted from pure cultures of *Xfm* were used as a template. To determine the optimal reaction temperature, LAMP was performed from 66 to 73 °C using 0.01 ng/µL of *Xfm* DNA sample diluted from the initially extracted DNA sample. The LAMP reaction was performed using Genie^®^ III (OptiGene) real-time amplification system using LavaLAMP™ DNA Master Mix (Lucigen) according to the reaction mixture and protocol described below (Section 4.10). The amplification effectivity of the sample at the above-stated temperature range using the Genie^®^ III system was assessed using two main parameters: amplification time (Ti_amp_) and amplicon annealing temperature (T_a_) [45]. The Genie^®^ III amplified product was also checked using agarose gel electrophoresis and visual observation was performed by adding 0.5 µL of SYBR™ Green I DNA gel staining agent under UV irradiation (Invitrogen). The optimum time for amplification was determined to be 60 min according to the previous studies [42,45,46].

### 4.9. Reaction Conditions of LAMP

The LAMP reaction was performed with LavaLAMP™ DNA Master Mix (Lucigen) in a 25 μL mixture containing 2.5 μL of primer mix, 12.5 μL 2× Lava LAMP™ DNA Master Mix, 1 μL Green Fluorescent Dye (Lucigen), 1 μL of DNA template, and nuclease-free H_2_O added up to the desired total volume. The 10× formulation for the regular LAMP was: 1.6 μM each of FIP and BIP; 0.2 μM each of F3 and B3; 0.8 μM each of LF and LB, respectively. By contrast, for FRET-based “probe LAMP” assay, in addition with 0.2 μM of F3 and B3 and 1.6 μM of FIP and BIP, the 10× formulation of the primer mix contained 0.1 μM of the quench (Q) strand and 0.2 μM of the FAM-tagged modified backward loop primer (or assimilating probe LB-F, Supplementary Figure S5, Supplementary Table S1) with 0.8 μM of LF primer. For LAMP amplification, the Genie^®^ III real-time amplification system was used and the data was analyzed using Genie explorer software (OptiGene). Genie^®^ III amplified products were further analyzed by 1% agarose gel electrophoresis for visual inspection and with SYBR™ Green 1 nucleic acid gel stain (Invitrogen) for naked-eye observation. The mixture was preheated at 90 °C for 3 min, amplified at 70 °C for 60 min, and then terminated at a range from 98 to 80 °C, with a decline rate of 0.05 °C per second.

### 4.10. Specificity Analysis of LAMP

The specificity of LAMP primers for the detection of *Xfm* was examined using 0.1 ng of DNA extracted from pure bacterial cultures of *Xfm* isolates AlmaReb3 and AlmaStar1 and *Xff* isolates AlmaReb1 and AlmaReb2. An additional specificity test was carried out with 7 isolates (3 *Xff* and 4 *Xfm*) from both subspecies (Table 2). The DNA extraction from pure culture and the LAMP assay were performed according to the methods described above (Section 4.3, Section 4.8, Section 4.9 and Section 4.10). Nuclease-free H_2_O was used as negative control.

### 4.11. Sensitivity Analysis of LAMP

The sensitivity of LAMP primers to amplify *Xfm* was examined using 10 ng/μL to 0.1 fg/μL of DNA extracted from *Xfm* isolate AlmaReb3. The assay was executed according to the method described above. Nuclease-free H_2_O was used as negative control.

### 4.12. Evaluation of Infected Field Samples Using CAPS Marker and LAMP Assay

Samples were taken from SHB blueberry plants with bacterial leaf scorch (BLS) symptoms from fields located in three different counties (Bacon, Pierce, and Ware counties) in the state of Georgia to detect and differentiate between the two subspecies of *Xf* infecting blueberry. Leaf samples were collected from at least five plants per site showing typical BLS symptoms (Table 1, Supplementary Figure S4). From each sample, total DNA was extracted from between three to five midribs and petioles of collected leaf material using a modified CTAB protocol (G-Biosciences, St. Louis, MO, USA). The *Xf*-positive samples were screened by polymerase chain reaction (PCR) testing using the RST31/33 primer pair [55]. Twenty-six *Xf* positive samples were selected for further CAPS based and probe-based LAMP analysis for subspecies identification according to the protocol described above. Subspecies identity was further confirmed by direct sequencing of the PCR product by Sanger sequencing.

## 5. Conclusions

BLS, which is caused by the two subspecies of *Xylella fastidiosa* (*Xf*), *X. fastidiosa* subsp. *Multiplex* (*Xfm*) and *X. fastidiosa* subsp. *Fastidiosa* (*Xff*) is a growing concern for blueberry producers in the southeastern United States. Robust methods for subspecies identification to investigate into the origins of isolates causing *Xf* outbreaks are indispensable. Unfortunately, the available detection techniques are unable to easily differentiate between the *Xff* and *Xfm* isolates infecting blueberry, and sequencing-based methods can be lengthy, costly, and relatively low throughput. Therefore, we developed two independent detection techniques: a CAPS marker and a LAMP assay to vigorously discriminate between the *Xfm* and *Xff* subspecies infecting blueberries. These methods were developed targeting either unique restriction sites or the in/del mutations and SNPs in RNA polymerase sigma-70 factor (*rpoD*) gene sequence of *Xfm* versus *Xff*. With these detection methods, *Xfm* was reliably detected and differentiated from pure culture grown, infected field-grown and inoculated greenhouse-grown *Xff* blueberry samples. Moreover, LAMP detection of *Xfm* (versus *Xff*) with three different approaches including gel electrophoresis, SYBR™ Green I DNA staining, or with Genie^®^ III allowed the detection method to be applicable in lab conditions and in the field. Taken together, our results demonstrate that both of our detection methods have the potential to rapidly and reliably enable differentiation between the *Xf* subspecies causing BLS in blueberry in the southeastern United States.

## Figures and Tables

**Table 1:** List of the *X. fastidiosa* infected blueberry field samples used in this study for subspecies detection and differentiation using CAPS marker and LAMP assay. Subspecies identity was further confirmed by direct sequencing of the RST PCR product.

Sample no.	SHB Cultivar	Year of Collection	Field Site ID	Geographic Location in Georgia	Subsp. Identity by CAPS Marker and LAMP Assay	Subsp. Identity by Direct Sequencing
FS1	Rebel	2019	Site 1	Bacon County	*Xfm*	*Xfm*
FS2	Rebel	2019	Site 1	Bacon County	*Xfm*	*Xfm*
FS3	Rebel	2019	Site 1	Bacon County	*Xfm*	*Xfm*
FS4	Rebel	2019	Site 1	Bacon County	*Xfm*	*Xfm*
FS5	Rebel	2019	Site 1	Bacon County	*Xfm*	*Xfm*
FS6	Rebel	2019	Site 2	Bacon County	*Xff*	*Xff*
FS7	Rebel	2019	Site 2	Bacon County	*Xfm*	*Xfm*
FS8	Rebel	2019	Site 2	Bacon County	*Xfm*	*Xfm*
FS9	Rebel	2019	Site 2	Bacon County	*Xfm*	*Xfm*
FS10	Rebel	2017	Site 2	Bacon County	*Xfm*	*Xfm*
FS11	Rebel	2018	Site 2	Bacon County	*Xff*	*Xff*
FS12	Rebel	2018	Site 1	Bacon County	*Xfm*	*Xfm*
FS13	Rebel	2018	Site 1	Bacon County	*Xfm*	*Xfm*
FS14	Rebel	2018	Site 1	Bacon County	*Xfm*	*Xfm*
FS15	Rebel	2017	Site 2	Bacon County	*Xff*	*Xff*
FS16	Rebel	2017	Site 2	Bacon County	*Xff*	*Xff*
FS17	Rebel	2017	Site 2	Bacon County	*Xfm*	*Xfm*
FS18	Star	2017	Site 3	Bacon County	*Xfm*	*Xfm*
FS19	Meadowlark	2017	Site 4	Pierce County	*Xff*	*Xff*
FS20	Sweet Crisp	2018	Site 5	Ware County	*Xfm*	*Xfm*
FS21	Rebel	2019	Site 1	Bacon County	*Xfm*	*Xfm*
FS22	Rebel	2019	Site 1	Bacon County	*Xfm*	*Xfm*
FS23	Rebel	2019	Site 1	Bacon County	*Xfm*	*Xfm*
FS24	Rebel	2019	Site 1	Bacon County	*Xfm*	*Xfm*
FS25	Rebel	2019	Site 1	Bacon County	*Xfm*	*Xfm*
FS26	Rebel	2019	Site 1	Bacon County	*Xfm*	*Xfm*

**Table 2:** Summary of *Xylella fastidiosa* isolates and sequences used in this study for in silico analyses and/or molecular detection assays.

Isolate Name	SHB Cultivar	Isolation Location	Subsp ID ^a^	Used for ^b^	Reference	RST Accession Number
PierceMed1	Meadowlark	Pierce County, GA, USA	*Xff*	*in silico* analyses and LAMP	Di Genova et al. [17]	MN590439
AlmaReb1	Rebel	Bacon County, GA, USA	*Xff*	*in silico* analyses, LAMP, and CAPS	Di Genova et al. [17]	MN590433
AlmaReb2	Rebel	Bacon County, GA, USA	*Xff*	*in silico* analyses, LAMP, and CAPS	Di Genova et al. [17]	MN590434
AlmaReb3	Rebel	Bacon County, GA, USA	*Xfm*	*in silico* analyses, LAMP, and CAPS	Di Genova et al. [17]	MN590435
AlmaStar1	Star	Bacon County, GA, USA	*Xfm*	*in silico* analyses, LAMP, and CAPS	Di Genova et al. [17]	MN590436
AlmaStar2	Star	Bacon County, GA, USA	*Xfm*	*in silico* analyses and LAMP	Di Genova et al. [17]	MN590437
AlmaStar3	Star	Bacon County, GA, USA	*Xfm*	*in silico* analyses and LAMP	Di Genova et al. [17]	MN590438
AlmaEm3	Emerald	Bacon County, GA, USA	*Xfm*	*in silico* analyses only	Oliver et al. 2014 [22]	PUIY01000010
BB01	V1	Brantley County, GA, USA	*Xfm*	*in silico* analyses only	Van Horn et al. 2017 [56]	MPAZ01000016
BB08-1	Windsor	Putnam County, FL, USA	*Xfm*	*in silico* analyses only	Oliver et al. 2014 [22]	PUIZ01000048
BBI64	V1	Brantley County, GA, USA	*Xfm*	*in silico* analyses only	Oliver et al. 2014 [22]	PUJA01000073

^a^*Xfm*: *Xylella fastidiosa* subsp. *multiplex*, *Xff*: *Xylella fastidiosa* subsp. *fastidiosa* ^b^. ”*in silico*” includes the analyses used to design the LAMP primers and CAPS marker, “LAMP” refers to the use of isolate DNA in the LAMP assay assessments, “CAPS” refers to the use of isolate DNA in the CAPS marker assessments.

## Supplementary Materials

The following are available online at https://www.mdpi.com/article/10.3390/ijms23041937/s1.
