# Supplementary material for: Development of a CAPS Marker and a LAMP Assay for Rapid Detection of Xylella fastidiosa Subsp. multiplex and Differentiation from X. fastidiosa Subsp. fastidiosa on Blueberry
Source: Int J Mol Sci. 2022 Feb 9;23(4):1937. doi: 10.3390/ijms23041937 (PMC8876805; doi:10.3390/ijms23041937)
Supplement: Supplementary file 1 [file ijms-23-01937-s001.zip › ijms-1536081-supplementary.pdf]

Supplementary Table S1. Oligonucleotide sequences used for PCR and LAMP in this study.

Assay	Primers	Sequence (5′-3′)	Product size (bp)	Reference
PCR	RST-31F	GCGTTAATTTTCGAAGTGATTCGATTGC	733	[55]
	RST-33R	CACCATTCGTATCCCGGTG		
Regular LAMP	F3	ATTCGATTGCTCCCTTGC	NA	This study
	B3	TGAATCAGAGCTGCTCATG		
	FIP	TCGTTGTTTGTTGCTTTTTGATCT*TTTTT*CCATGTAAACCCATTTCACTAG		
	BIP	CAGTGCCGCATCAACATCGTA*TTTTT*CTTTCCCATACGTGCTTAA		
	LF	AAGAGGGGGCTGATCTTTG		
	LB	CTAGATCACATTTGCTTGCCCTAC		
Probe-based LAMP	F3	ATTCGATTGCTCCCTTGC	NA	This study
	B3	TGAATCAGAGCTGCTCATG		
	FIP	TCGTTGTTTGTTGCTTTTTGATCT*TTTTT*CCATGTAAACCCATTTCACTAG		
	BIP	CAGTGCCGCATCAACATCGTA*TTTTT*CTTTCCCATACGTGCTTAA		
	LF	AAGAGGGGGCTGATCTTTG		
	Assimilating probe* LB-FAM, florescent strand	FAM-ACGCTGAGGACCCGGATGCGAATGCGGATGCGGATGCCGA CTAGATCACATTTGCTTGCCCTAC	NA	This Study
	Q, quench strand	TCGGCATCCGCATCCGCATTCGCATCCGGGTCCTCAGCGT-BHQ		

*Assimilating probe was designed according to Kubota et al. (2011). Underlined fragment is the backward loop primer (LB) from the regular LAMP assay. FAM is 6-carboxyfluorescein and BHQ is Black Hole Quencher.

Supplementary Table S2. LAMP temperature optimization using the real-time LAMP detection system Genie® III. Selected temperature for LAMP amplification is highlighted in bold.

Tm_{amp} (°C)[a]	Ti_{amp} (Min:S)[b]	Ta (°C)[c]
66	37:15	83.0
67	34:00	83.0
68	31:15	82.9
69	30:00	83.0
**70**	**24:30**	**83.6**
71	28:00	83.2
72	00:00	83.9
73	02:00	83.3

[a]Amplification temperature in degree Celsius (°C)
[b]Amplification time in minutes and seconds (Min:S)
[c]Annealing temperature in degree Celsius (°C)

Supplementary Table S3. Sensitivity analysis of LAMP using the real-time LAMP detection system Genie® III. The marginal nucleic acid concentration for true LAMP amplification is highlighted in bold.

Name of well	C_{amp} (pg/µl)[a]	Ti_{amp} (Min:S)[b]
1	100	28:30
2	10	32:15
**3**	**1**	**35:30**
4	0.1	55:00
5	0.01	56:15
6	0.001	N/A
7	0.0001	N/A
Neg	0	N/A

[a]*Xylella fastidiosa* subsp. *multiplex* DNA concentration in pg/µl
[b]Amplification time in minutes and seconds (Min:S)

Supplementary Table S4. Probe-based LAMP temperature optimization using the real-time LAMP detection system Genie® III. Selected temperature for LAMP amplification is highlighted in bold.

Tm_{amp} (°C)[a]	Ti_{amp} (Min:S)[b]
66	30:15
67	27:45
68	25:15
69	24:00
**70**	**23:45**
71	26:00
72	00:00
73	00:00

[a]Amplification temperature in degree Celsius (°C)
[b]Amplification time in minutes and seconds (Min:S)

Supplementary Table S5. Sensitivity analysis of probe-based LAMP using the real-time LAMP detection system Genie® III. The marginal nucleic acid concentration for true LAMP amplification is highlighted in bold.

Name of well	C_{amp} (pg/µl)[a]	Ti_{amp} (Min:S)[b]
1	100	31:15
**2**	**10**	**34:15**
3	1	46:15
4	0.1	54:30
5	0.01	N/A
6	0.001	N/A
7	0.0001	N/A
Neg	0	N/A

[a]*Xylella fastidiosa* subsp. *multiplex* DNA concentration in pg/µl
[b]Amplification time in minutes and seconds (Min:S)

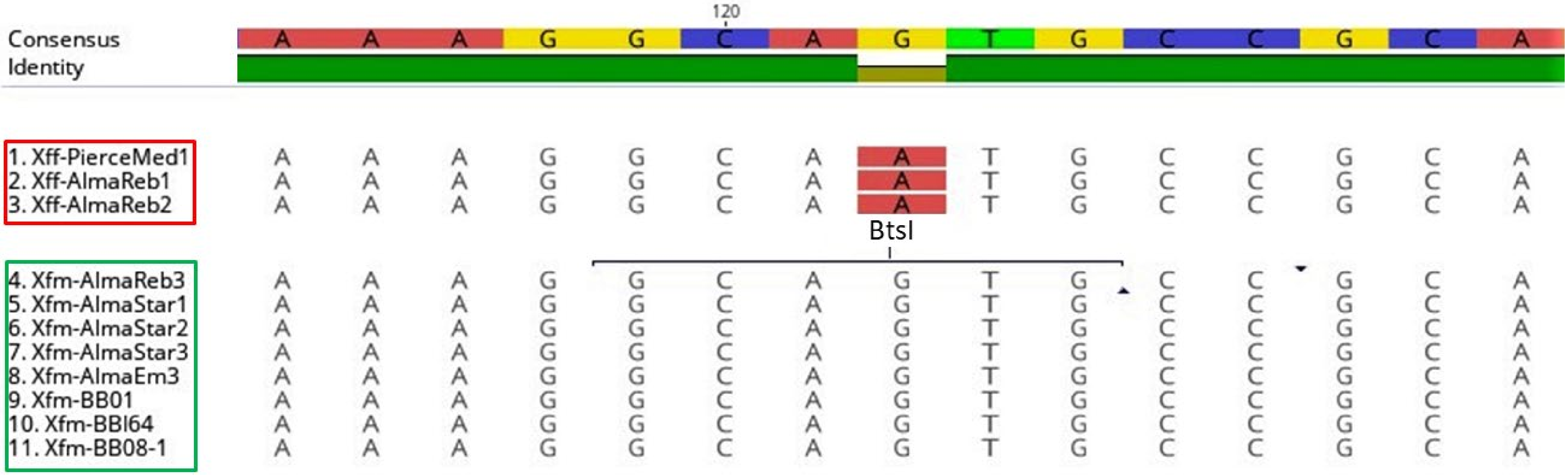

Supplementary Figure S1. Partial *rpoD* sequences showing the *BtsI* restriction site in aligned DNA of *X. fastidiosa* subsp. *multiplex* and *X. fastidiosa* subsp. *fastidiosa* isolates from blueberry. All publicly available *rpoD* sequences for *X. fastidiosa* isolates from blueberry were utilized including three *X. fastidiosa* subsp. *fastidiosa* (*Xff*) isolates and eight *X. fastidiosa* subsp. *multiplex* (*Xfm*) isolates. The red box indicates *X. fastidiosa* subsp. *fastidiosa* isolates and the green box indicates *X. fastidiosa* subsp. *multiplex* isolates.

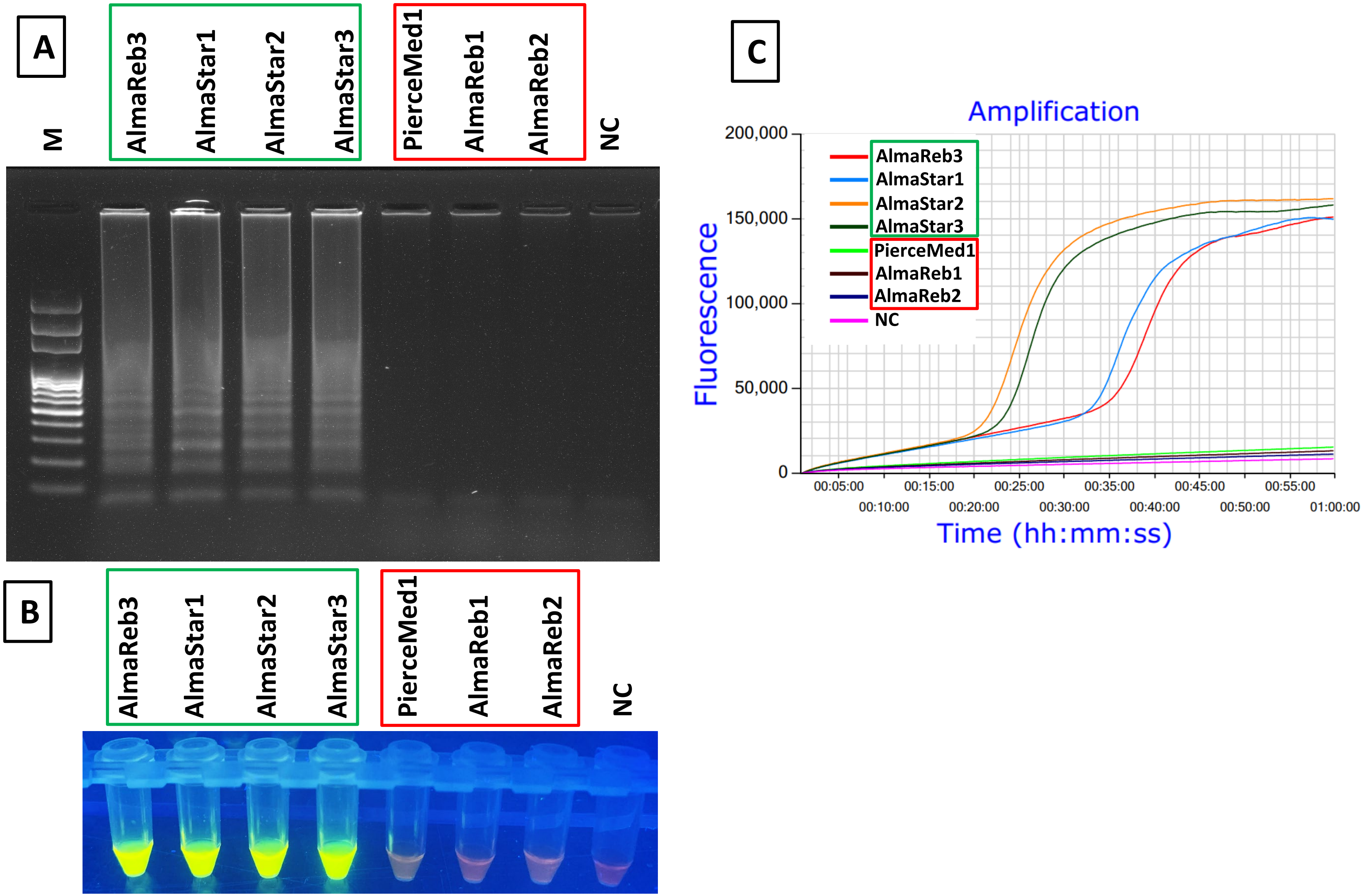

Supplementary Figure S2. Specificity confirmation of the LAMP assay for the detection of *X. fastidiosa* subsp. *multiplex* from DNA from known bacterial cultures. Amplified LAMP products were analyzed using: A. agarose gel electrophoresis, B. visual inspection with SYBR™ green 1 DNA gel staining, and C. real-time amplification using Genie® III. M = 100 bp DNA ladder, NC = nuclease-free H_2O as a negative control. The fluorescent green-colored products for each *X. fastidiosa* subsp. *multiplex* isolate, excluding the negative control, could be visualized after SYBR™ green 1 DNA gel staining under UV light. The red boxes indicate *X. fastidiosa* subsp. *fastidiosa* isolates and the green boxes indicate *X. fastidiosa* subsp. *multiplex* isolates.

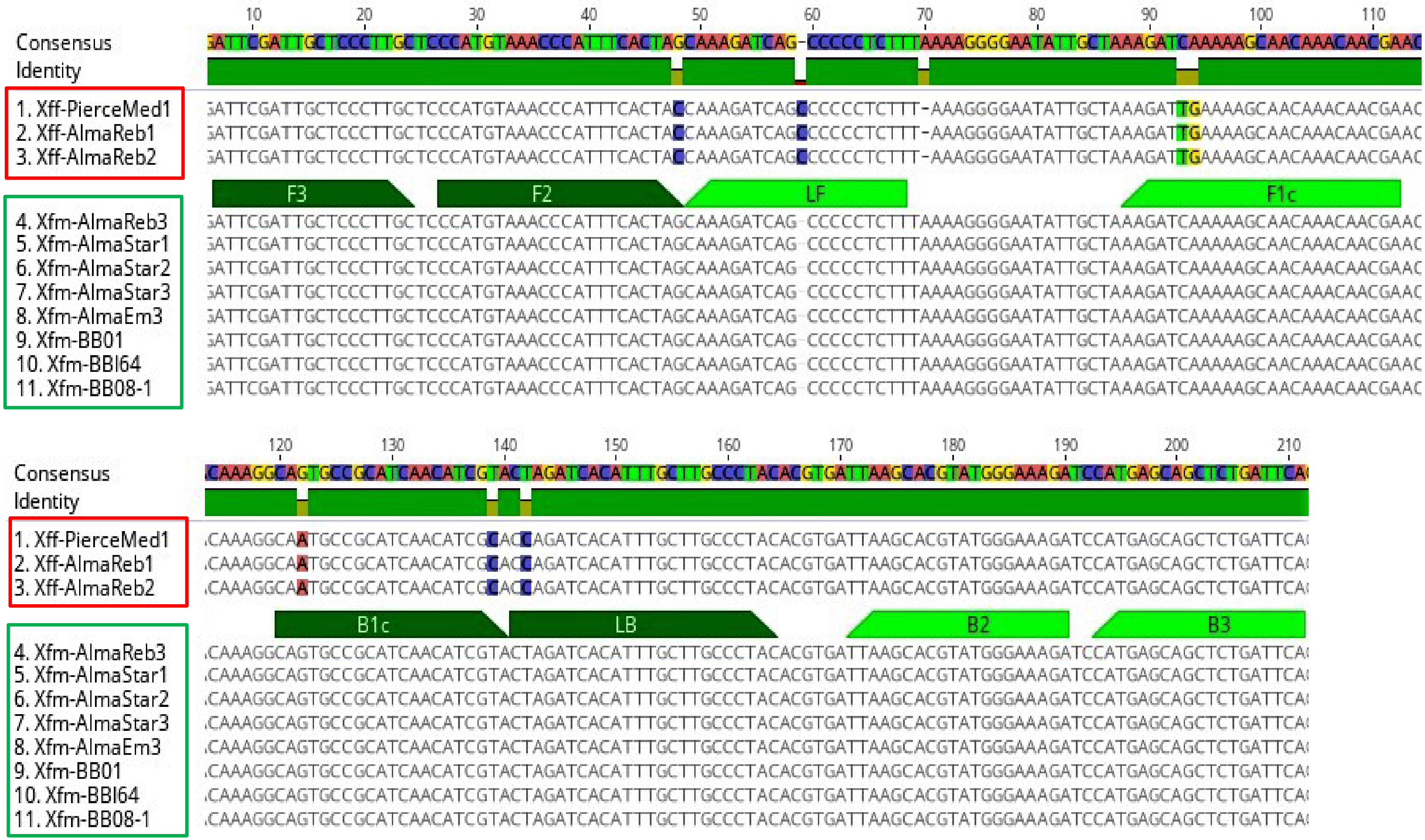

Supplementary Figure S3. An alignment showing single-nucleotide polymorphisms (SNPs), insertion and deletion mutations (colored letters) for *X. fastidiosa* subsp. *multiplex* LAMP primer binding sites versus *X. fastidiosa* subsp. *fastidiosa* within the isolates' *rpoD* sequences. The red boxes indicate *X. fastidiosa* subsp. *fastidiosa* isolates and the green boxes indicate *X. fastidiosa* subsp. *multiplex* isolates. All publicly available *rpoD* sequences for *X. fastidiosa* isolates from blueberry were utilized including three *X. fastidiosa* subsp. *fastidiosa* (*Xff*) isolates and eight *X. fastidiosa* subsp. *multiplex* (*Xfm*) isolates.

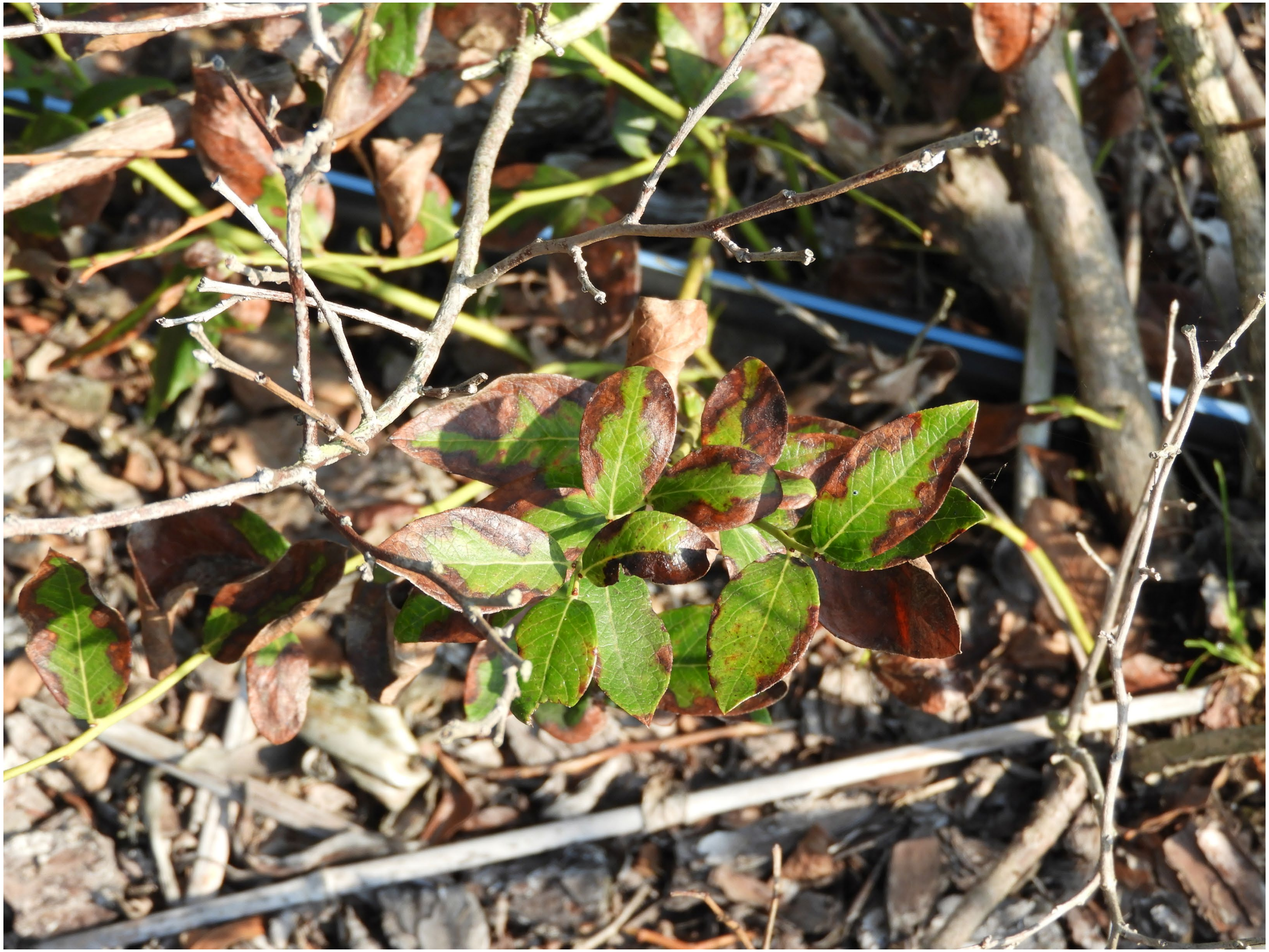

Supplementary Figure S4. Photograph showing a symptomatic *X. fastidiosa*-infected SHB blueberry field plant sampled in this study for molecular analysis. The infected leaves show typical BLS disease symptoms.

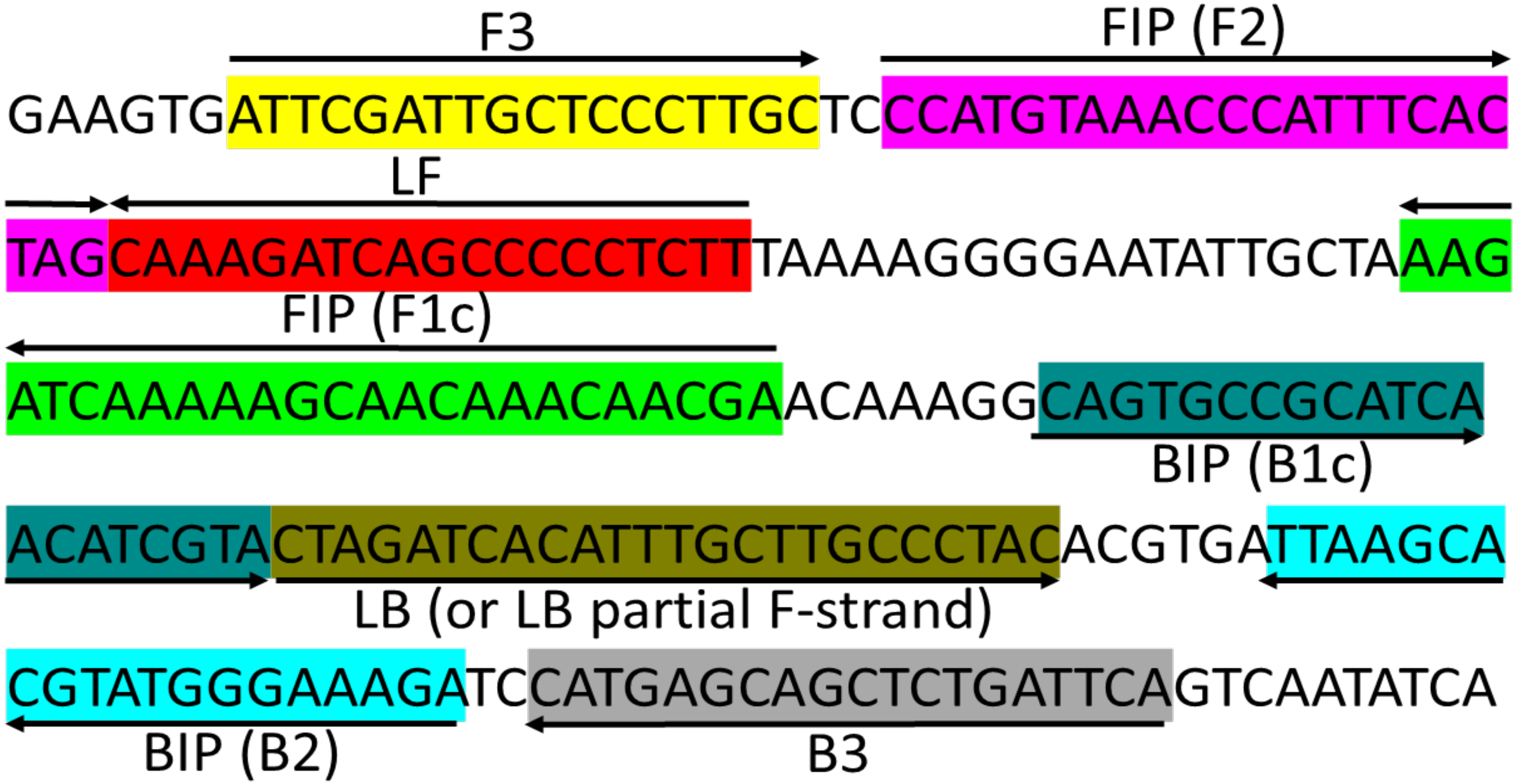

Supplementary Figure S5. Location and partial sequence of loop-mediated isothermal amplification (LAMP) primer (or probe) sets targeting *X. fastidiosa* subsp. *multiplex* specific DNA. Primer (or probe) locations for LAMP assay are indicated by black arrows (F3, B3, FIP [F1c-F2], and BIP [B1c-B2]). FIP is a hybrid primer consisting of the F1c sequence and the F2 sequence, BIP is a hybrid primer consisting of the B1c sequence and the B2 sequence. Backward loop primer was targeted to create an F-strand for probe-based LAMP detection. Arrows indicate the extension direction.

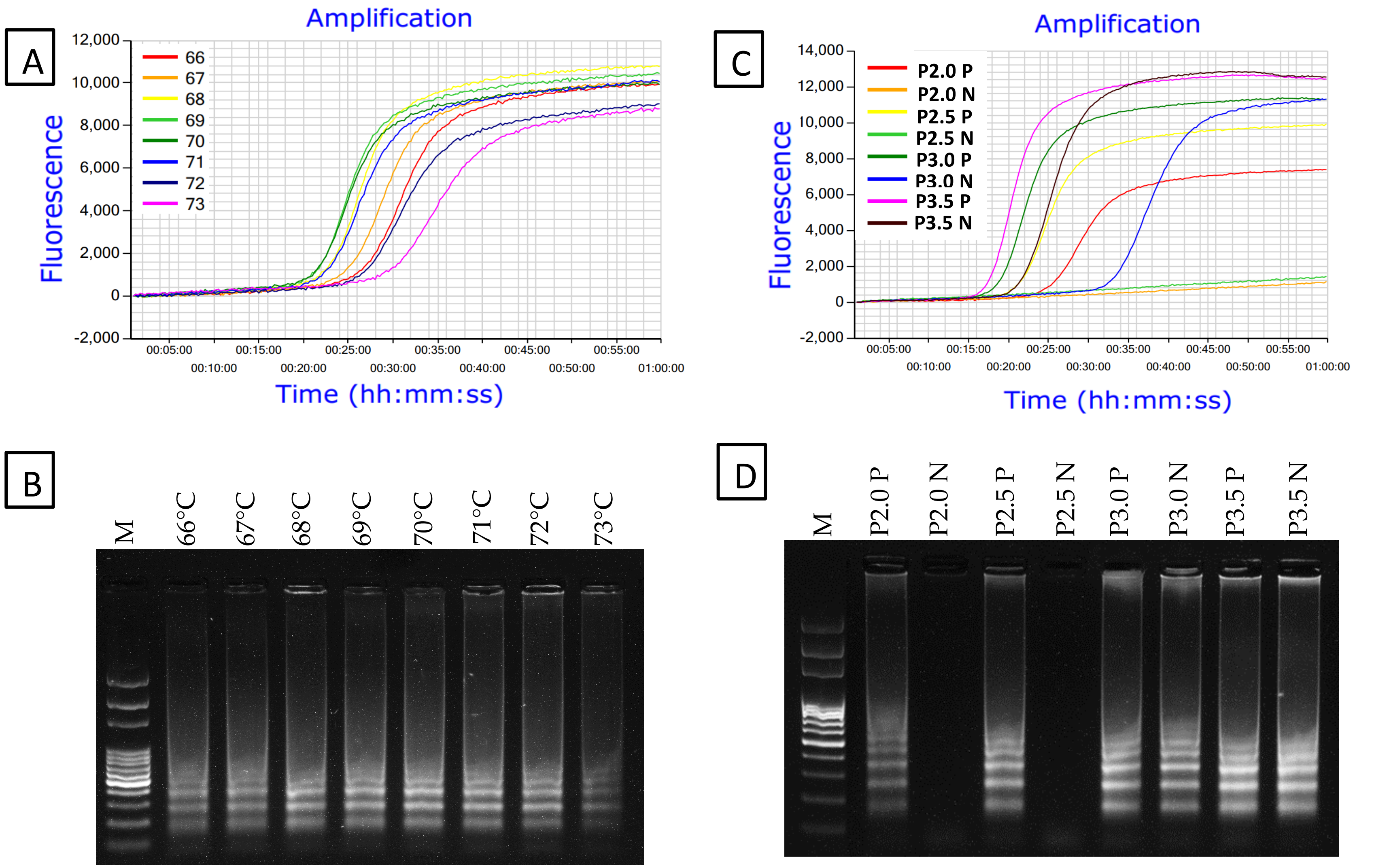

Supplementary Figure S6. Optimization of reaction conditions of *X. fastidiosa subsp. multiplex* probe-based LAMP. Results were analyzed by real-time amplification with Genie® III (A, C) and agarose gel electrophoresis (B, D). For temperature optimization (A, B), a gradient LAMP was set using a temperature range from 66 to 73°C. And for the optimization of primer/probe concentration (C, D), four separate primer/probe mixture volumes (2.0, 2.5, 3.0 and 3.5 µl) were tested with a positive (Xfm isolate AlmaReb3) and negative (dH20) controls (P and N). Here, M = 100 bp DNA ladder.

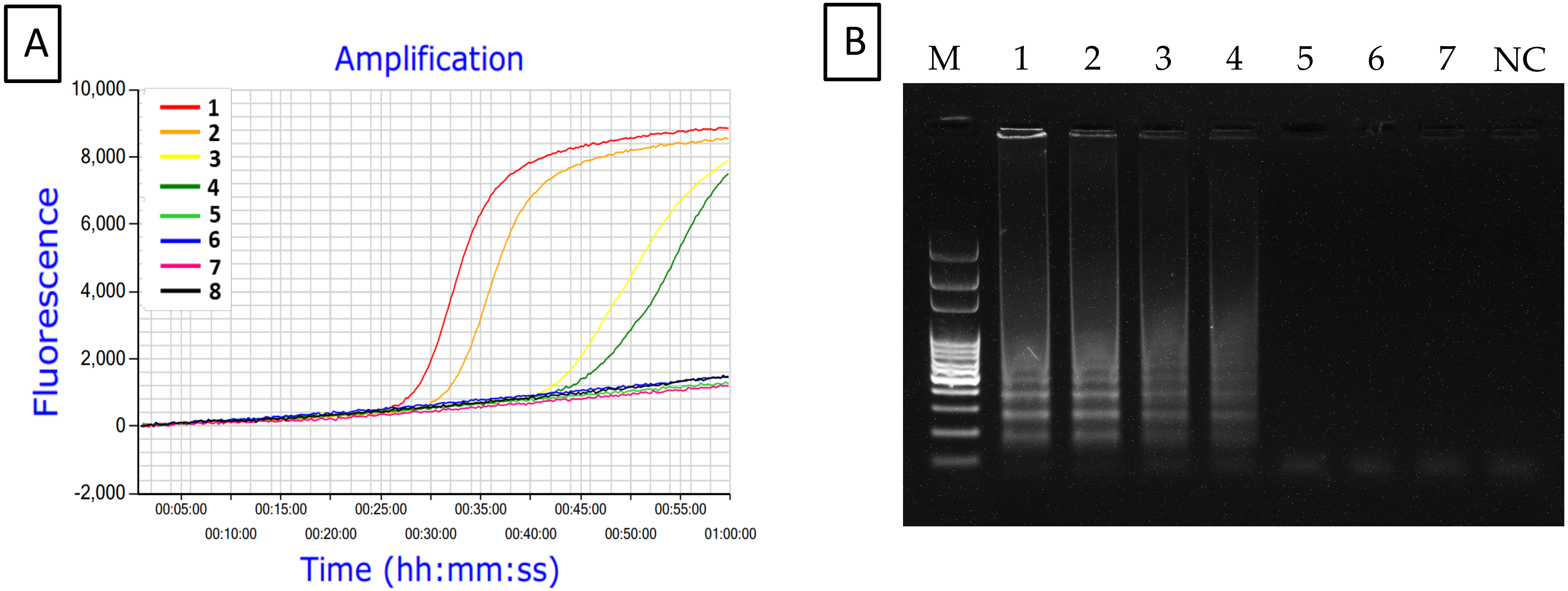

Supplementary Figure S7. Sensitivity of the probe-based LAMP assay for the detection of *X. fastidiosa* subsp. *multiplex* using DNA from Xfm isolate AlmaStar2.1. Amplified LAMP products were analyzed using: A. real-time amplification using Genie® III and B. agarose gel electrophoresis. Here, 1 to 7: 100pg/µl to 0.0001pg/µl of Xfm DNA. M = 100 bp DNA ladder, NC = nuclease-free H2O as a negative control.
